# Reaching the Unreached: Unmet Needs and the Promise of Telehealth Among People with Mobility Disabilities in Low-Resource Areas in Alabama

**DOI:** 10.3390/disabilities6020040

**Published:** 2026-04-17

**Authors:** James Rimmer, Victoria Christian, Raven Young, Stephanie Ward, Pooja Arora, Phuong Quach, Byron Lai

**Affiliations:** 1 Dean’s Office, School of Health Professions, University of Alabama at Birmingham, Birmingham, AL 35294, USA;; 2 Department of Physical Medicine and Rehabilitation, University of Alabama at Birmingham, Birmingham, AL 35294, USA; 3 Division of Pediatric Rehabilitation Medicine, Department of Pediatrics, University of Alabama at Birmingham, Birmingham, AL 35294, USA;; 4 Department of Physical Therapy, School of Health Professions, University of Alabama at Birmingham, Birmingham, AL 35294, USA;

**Keywords:** community, mental health, poverty, mobility disability, barriers

## Abstract

**Background:**

Adults with disabilities living in low-resource communities experience persistent inequities in access to healthcare, mental health services, and community participation. However, qualitative data capturing lived experiences in the Deep South remain limited. This study aimed to identify priority needs among adults with mobility disabilities residing in economically distressed communities near Birmingham, Alabama, to inform future telehealth programming.

**Methods:**

Fifteen adults (mean age = 60 ± 10 years), predominantly African American and female, completed semi-structured phone interviews exploring basic needs, neighborhood accessibility, health priorities, and perceived supports. Interviews were audio-recorded, transcribed verbatim, and analyzed using Braun and Clarke’s six-phase thematic analysis.

**Results:**

Five themes emerged: (1) seeking stability amid severe mental health strain and inadequate supports; (2) constrained food environments shaped by cost, location, and safety; (3) feeling forgotten: systemic neglect and restricted participation in community life; (4) physical health deprioritized by competing needs and structural barriers; and (5) remote support as a viable but unrealized option. Participants described how safety concerns, transportation barriers, and rising food costs constrained daily functioning, while unmet mental health needs compounded isolation. Despite widespread cardiometabolic disease, immediate needs related to mental health, food, and housing consistently superseded physical health. Mental health support was identified as the most feasible area for remote delivery, though poor awareness of available resources limited engagement with any service model.

**Conclusions:**

Findings demonstrate that disability-related disparities in low-resource communities are driven largely by structural and environmental factors rather than individual choice. Telehealth and mobile-based services may provide a feasible access strategy for mental health and supportive care in under-resourced settings, particularly when integrated with broader community supports. Addressing foundational needs is essential for advancing health equity among people with disabilities in the Southeast.

## 1. Introduction

In the United States, 20.9% of people with a disability live in poverty and often lack the necessary resources to meet their healthcare needs [1]. The cost of medical care associated with disability, including equipment and the management of potential comorbidities, also drives up the average cost of living for these individuals [2]. Inequities in healthcare are evident through delayed care, reduced access to preventive services, and overall poorer health outcomes [3,4]. Nationally, adults with activity limitations are significantly more likely to postpone care due to cost and financial burden and are four times more likely to have emergency department visits and hospitalizations [4–6]. The shortage of healthcare providers in rural counties is also a significant barrier to accessing routine services [7,8]. Because of limited resources, families often need to arrange transportation and take time off work to attend medical appointments [1].

The Southeast region has a higher prevalence of mental distress compared to the national average, a pattern that parallels the region’s elevated rate of disability [9]. Compared to other regions of the United States, the South has a higher prevalence of people living with disability, with median county-level rates of 29.5% [7,8]. People with disabilities in the Southeast experience healthcare disparities driven by socioeconomic and systemic factors, contributing to poorer long-term health outcomes [2]. Indeed, within the South, the term “doubly disadvantaged” describes individuals with disabilities who are more likely to live in low-resource, rural communities or belong to racial or ethnic minority groups [4,5].

Most research describing the health issues and living arrangements of people with disabilities relies on survey-based methods. While these studies are valuable for large-scale comparisons across states, regions, and populations, they offer limited insight into the lived experiences of people with disabilities. Relatively few qualitative studies have been conducted to capture these deeper perspectives. This gap is particularly pronounced in the Deep South, where structural barriers, including high rates of poverty, crime, and limited community resources, make it difficult to access and engage people with disabilities living in under-resourced communities. Accordingly, the purpose of this qualitative study is to center the voices of people with mobility disabilities in one Deep South city and surrounding region (Birmingham, Alabama) to identify both basic and health-related needs in economically distressed areas of the Southeast, with the goal of informing future telehealth programming.

## 2. Materials and Methods

This qualitative study was underpinned by the following philosophical assumptions: relativism ontology [10] and interpretivism epistemology [11]. These views acknowledge that participants hold diverse and subjective realities, while recognizing that knowledge is constructed by the interaction between the participant and researcher.

### 2.1. Participants

Participants resided in economically distressed communities in the Birmingham metropolitan area that are designated as high-poverty census tracts, defined as census tracts with poverty rates of 20% or more per federal poverty area measures, or areas that align with certain federal designations such as Qualified Opportunity Zones, reflecting persistent structural disadvantage in income, investment, and access [12,13]. To be included in the study, participants had to have an observable (identified through in-person recruitment efforts) or self-reported mobility disability and reside within a designated low-resource area. Participants were considered to have a mobility disability if they had difficulty walking or climbing stairs, or used an assistive device such as a cane, walker, or wheelchair. This includes difficulty walking about a quarter mile (roughly 2–3 city blocks) or climbing a flight of stairs without help (U.S. Census Bureau, *American Community Survey*) [14].

The study team aimed to enroll a convenience sample of 20 participants, with a minimum goal of 12, which was deemed to be sufficient based upon relevant, previous work involving community leaders [15]. Participants were recruited through word-of-mouth referrals from community leaders (elected neighborhood representatives serving rotating terms of one to two years), from the database of the National Center on Health, Physical Activity, and Disability (NCHPAD, which is funded by the Centers for Disease Control and Prevention and headquartered in Birmingham, AL, USA), and in person at a hospital in Birmingham that serves uninsured and underinsured county residents. This study was determined to be “not research” by the University Institutional Review Board, and verbal consent was obtained from all participants prior to their involvement. The first participants to respond to recruitment efforts were selected to be included in the study.

### 2.2. Procedures

Participants completed the study in a single phone call interview. Phone calls included study briefing, verbal consent, and a semi-structured interview. The interview included 10 questions aimed at exploring basic needs, with an emphasis on how needs could potentially be addressed through telehealth services. Interviews began with an introductory icebreaker to build rapport and gather background information about participants and their daily activities (Question 1). Participants were then asked to reflect on their overall health (Question 2) and to describe perceived opportunities within their community that support physical (Question 3), mental (Question 4), and spiritual well-being (Question 5). Following questions explored whether participants felt their basic needs were met (Question 6) and whether their neighborhood environment was accessible, including their ability to navigate and participate comfortably within the community (Question 7). Accessibility was intentionally left undefined during interviews to allow participants to interpret the term according to their own lived experience. Participants were invited to discuss perceived gaps in accessibility (Question 8), inclusion in neighborhood activities (Question 9), and potential strategies to improve engagement and support (Question 10). Interviews concluded with an open-ended opportunity for participants to share any additional perspectives or experiences not addressed by the listed questions. Interview questions were followed up by additional questions to probe for greater details and deeper meaning, particularly related to how telehealth could potentially address identified needs. Interviews were audio-recorded so that they could be transcribed for analysis. Participants were compensated with a $20 electronic Amazon gift card that was emailed to them after completing the phone call.

### 2.3. Analysis

The interviews were conducted by two interviewers (B.L. and R.Y.). One interviewer (B.L.) had several years of experience interviewing greater than 500 people with disabilities. The second interviewer (R.Y.) was a disability exercise research coordinator with several years of experience in working with people with disabilities in clinical trials and settings. These interviewers also analyzed the data.

Using Braun and Clarke’s six-phase thematic analysis approach, a qualitative analysis was performed of the interviews as seen in Table 1 [16,17]. Audio recordings were transcribed by two team members (P.Q. and P.A.). Analysis began with two authors (B.L. and R.Y.) familiarizing themselves with the transcriptions enough to develop general themes and patterns within the results. The transcripts were then coded (done manually by the two analysts with no software used), with each code helping to identify instances that display moments of meaning, emotion, perceptions, or action. These codes were then organized into themes that were apparent across the dataset. A general rule for the analysts was to ensure that themes were not overtly specific, given the small sample size and only a single brief interview, to prevent more specific sub-categories from becoming less generalizable. The themes were then reviewed by the two analysts and revised to ensure they addressed the study’s purpose effectively while remaining distinct concepts. Quotes within the transcripts were extracted to further strengthen the support for the themes. The analytic process was iterative, incorporating ongoing reflexive consideration by the research team.

## 3. Results

A total of 15 people with a mean age of 60 ± 10 yrs from low-resource or rural areas completed the interview. Participants were predominantly female (13 out of 15) and African American (12 out of 15) and included a range of disabilities and conditions. Characteristics per participant are shown in Table 1. The thematic analysis yielded five overarching themes, each with embedded sub-themes that together reflect the layered structural and personal forces shaping participants’ lived experiences. The resultant themes are described below, with supporting codes and representative participant quotes provided in Supplementary File S1.

### 3.1. Theme 1: Seeking Stability Amid Severe Mental Health Strain and Inadequate Supports

Mental health emerged as the most salient and frequently discussed dimension of wellbeing across participants. Participants were heavily burdened by a variety of conditions that they navigated without formal support, such as depression, anxiety, bipolar disorder, and chronic emotional distress. Their lived experience with maintaining mental health was described as a constant battle: weighted by social isolation and a lack of professional support, but held afloat by strong links to spirituality within their community.

Within this overarching theme, two intersecting sub-themes were identified. The first concerned mental health decline in the absence of reliable social support. Participants described how the compounding effects of physical limitation, pain, deaths of loved ones or friends, and social disconnection created conditions in which mental health steadily eroded. One participant described a period of profound withdrawal: “*I just don’t have any social life. So I cut everybody out of my life. I was way too emotional for the first year. I couldn’t even speak without crying*” (R1). Social networks were small and fragile. Many participants did not maintain strong family connections and neighborhood communications were largely deemed unsafe due to violence and crime. Another participant connected present-day mental health struggles to unresolved childhood experiences: “*mental health, you know, they have not, what is it, healed that inner child or whatever it is*… *it stems from the way we grew up. And if you have not healed from whatever it is, whatever trauma or*… *dysfunction, it’ll manifest in your adulthood, good, bad or ugly*” (R3). This account captures mental health as not a discrete short-term event, but instead as a lived experience that is embedded in the biographical and relational history of the person.

The second sub-theme concerned mental health as a severe issue worsened by the lack of professional support. Across participants, depression and bipolar disorder were common and constantly stressful, yet access to counseling was described as either unavailable or prohibitively expensive. Participants expressed this gap with urgency: “*they don’t offer no recreation support groups to basically support my mental health. There’s not no support group out there for people that are like me, that have to deal with these problems every day*” (R2). One participant reported that their doctor would not refer them to a mental health professional, despite multiple pleas for help over longer than a year for assistance to cope with the loss of their loved one. Indeed, mental health services were urgently desired, with a need for the medical system and health professionals to understand the problem from those living inside it: “*I wish that there was more resources for mental illness*… *Put yourself in our shoes and see what exactly we go through*” (R2). Stress related to community safety further compounded mental health burden, as participants described ongoing anxiety about violence, theft, and unpredictable neighbors. One participant reflected that socialization in community settings required careful, guarded engagement: “*sometimes community things*… *you let people in just a little bit and they take you*… *it is kind of tough to trust people around your house or in community settings*” (R6). The same participant reported an instance where items on their property were stolen, shortly after inviting new people from the neighborhood into their home.

A third sub-theme, spirituality as an abundant but insufficient buffer for wellbeing, captured the way in which faith and church participation provided meaningful resilience and social connection, but could not fully substitute for professional mental health care. Churches were described as abundant, culturally embedded, and physically accessible: “*It’s churches on every corner*… *I’m quite sure if I wanted to, it would meet my spiritual needs*” (R7). Faith offered an interpretive framework through which participants made sense of suffering: “*as long as you put God first, everything will be all right*” (R3). Yet the same participants who drew comfort from spirituality also articulated the limits of faith-based support in addressing clinical mental health needs, underscoring the view that individual religious resources and formal structural supports serve distinct, non-interchangeable functions. On a positive note, all participants strongly emphasized that their spiritual needs were being met through regular participation in either in-person (nearby) or digital church congregations.

### 3.2. Theme 2: Constrained Food Environments Shaped by Cost, Location, and Safety

Food insecurity was woven through participants’ accounts of daily life, experienced not as a singular problem but as the cumulative product of structural forces: rising food costs, limited proximity to quality groceries, inconsistent public assistance, and physical and safety-related barriers to neighborhood navigation. Participants did not lack awareness that healthier eating was desirable; rather, their accounts made clear that for those subsisting largely on monthly disability support, healthier eating remained an aspiration the surrounding systems had made effectively unreachable.

Government food support programs were a necessary resource for many, though the quality and consistency of that support varied considerably. One participant described receiving food from a local bank that was entirely outdated—describing spoiled produce and expired goods offered as if normal: “*they gave me a key lime pie*… *it was nearly a year outdated*… *the cabbage was black on the inside*” (R4). Others were unable to locate food assistance resources at all, despite actively seeking them: “*I haven’t seen any*… *I don’t see any type of food vans or anything like that, meals for wheels or nothing like that for senior citizens coming through*” (R5). In contrast, some participants described food banks as genuinely helpful community assets, reflecting the diversity of subjective experience across participants and reinforcing the study’s relativist framing—that there was no single, uniform reality of food access, but multiple truths shaped by specific locations, individual resourcefulness, and the luck of proximity.

The rising cost of grocery store food was a second sub-theme, with participants describing healthy eating as effectively priced out of reach. For many, the Supplemental Nutrition Assistance Program (SNAP) provided some government-supported relief. However, benefits were widely perceived as insufficient to cover the cost of fresh, nutritious food, leaving participants to stretch limited allotments toward food options chosen primarily for their cost-effectiveness, with satiety and shelf life taking precedence over nutritional quality. Major grocery stores were perceived as not affordable and local small retailers were non-existent or non-sustainable. Dollar stores were identified as ideal for cost-effectiveness. Nevertheless, the cost of food was compounded by difficulties in allocating transportation. Without a personal vehicle or reliable public transit, food options were limited to what was nearest. Participants also described the physical environment as a barrier to walking to food sources, with no sidewalks, pothole-riddled roads, and road conditions that were described as genuinely dangerous for people with mobility impairments: “*if you get people like me with*… *mobility*… *disability, it can really, really hurt me if I come to those situations*” (R6). The cumulative picture that emerges from this theme is one in which food insecurity is not a behavioral outcome but a structural one—a convergence of cost, geography, transportation, and physical access that constrains what choices are realistically available.

### 3.3. Theme 3: Feeling Forgotten: Systemic Neglect and Restricted Participation in Community Life

A pervasive sense of being deprioritized by external systems ran through participants’ accounts of their community environments, neighborhood governance, and health services. When discussing needs, participants consistently positioned themselves as peripheral to the city’s concerns: “*I think people forget about us up here on this hill*… *they just forget about us*” (R7). This feeling was echoed through several community experiences, such as unkept vegetation requiring repeated calls to the city, garbage left uncollected, and emergency services that participants felt did not adequately serve their neighborhood. Police presence was described as insufficient, with participants noting that safety concerns went largely unaddressed despite repeated attempts to reach local officials. The fire service fared no better in participants’ accounts. One participant described a neighbor’s house fire where crews ran hoses the length of the street because they did not know a hydrant was nearby, concluding: “*they need to make sure that this little area up here*… *it’s not forgotten about because I feel it here*” (R7). The phrase ‘*I feel it here*’ is worth pausing on. Neglect, in this context, was not simply a logistical inconvenience. It was something participants carried with them.

Communal neglect was compounded by an unsafe neighborhood that restricted outdoor participation. Participants described a neighborhood environment shaped by poverty, where desperation and crime limited the ability to engage with neighbors and local spaces. Trust due to safety or theft was a recurring issue, which impeded community involvement, as described by one participant:

“*Sometimes community things, you know. You let people in just a little bit, and they take you. Give them a little inch and take them out. So it is kind of tough to trust people around your house or in community settings*”

(R6).

Another linked this directly to unmet need: “*a lot of times people like when things ain’t going their way, they should just go steal, rob, hurt somebody or whatever*” (R6). Participants also described loose and aggressive dogs as a genuine physical threat, particularly for those with mobility impairments who could not move quickly to avoid them: “*you really can’t enjoy like, around the block walk, because*… *dogs come and they want to take over the neighborhood*” (R6). Violence compounded this. One participant had stopped visiting a nearby park entirely, noting: “*they always got something going on if somebody getting shot or something. And I just don’t feel safe*” (R7). The built environment created additional safety barriers. Participants described neighborhoods with no sidewalks, high porch steps, and fast-moving vehicles on one-way streets with no speed control: “*we don’t have sidewalks*… *we need a couple of speed bumps cause*… *some of these youngsters come up through here, they drive so fast*” (R6). Another noted simply: “*all the porches are high. The steps are deep*” (R5). Due to these conditions, community spaces that might otherwise support social connection and physical activity were effectively off-limits.

The neglect participants described in their neighborhoods extended into how they experienced their health-related needs. Whether participants were describing the absence of mental health counseling, the cost of food, barriers to transportation, or the difficulty of accessing health care, a common thread ran through their accounts: that the systems meant to serve them had largely passed them by. Across themes, this sense of being passed over was less a standalone finding and more a consistent feature of daily life.

### 3.4. Theme 4: Physical Health Deprioritized by Competing Needs and Structural Barriers

Physical health was a substantial and daily burden for most participants. Pain and cardiometabolic conditions such as hypertension and diabetes were common, and participants described how these conditions directly limited their ability to be active and participate in the community: “*because they can’t get my sugar under control, my blood pressure stays high*” (R4), and “*my diabetes, if it’s a certain temperature, I will pass out. I can’t take the heat*” (R2). Yet despite this burden, physical health was rarely identified as a priority need. Healthcare costs, housing, and food insecurity were often the primary discussion topics, which structural barriers made difficult to address. Transportation limited access to health-related visits and exercise programs. Community spaces for physical activity were either absent or inaccessible: “*there’s no parks*… *I had to drive miles to find a greenway*… *it’s not taken care of very well*” (R1). Local exercise and health promotion resources were described as inadequate and poorly matched to the needs of working-age adults with disabilities: “*they don’t have nobody to really target the young people like me that are disabled*” (R2). Even acquiring basic assistive devices was financially out of reach: “*I’ve been trying to get help getting $300 for some hearing aids. And I was having to pay what money my husband brings in to first have a roof over our heads*… *And I don’t have $300 for no hearing aids*” (R4). One participant captured the broader issue directly: “*we need insurance for our body*… *make it a priority*… *why can’t we have mandatory exercise?*” (R3). Thus, physical health was not deprioritized by choice. When transportation barriers, cost, and the lack of local resources restricted access to even the most basic needs, physical health was what remained out of reach.

### 3.5. Theme 5: Remote Support as a Viable but Unrealized Option: Mental Health, Transportation, and the Information Gap

Across the four prior themes, a consistent pattern emerged in which participants identified significant unmet needs but faced structural barriers that made in-person services difficult or impossible to access. When asked how needs might be addressed through remote or technology-assisted delivery, mental health support was the most readily identified area. Participants who could not access counseling due to cost or transportation described wanting someone to talk to, and several were already engaging with remote programming in some form: “*I’m signed up in a program now that helps with the exercise and mobility and food health and all that stuff”* (R15), while another described virtual group participation as a workable substitute for in-person contact: “*I like group meetings. I do one now, but it’s on Zoom. I prefer to do something in person and I haven’t been able to find anything like that*” (R7). A recurring barrier to engagement with any service model, however, was not willingness but awareness. Participants described consistently not knowing what resources existed: “*there’s no information*… *if someone would come around and give flyers and talk to people about their overall health, I think they’d get a better response*” (R12). For participants with internet access, remote delivery of physical health or food-related telehealth services appeared feasible and interesting, but in-depth feedback was limited by unrealized options: “*I do have strong internet access*… *I would be interested in after my procedure*… *seeing about y’all’s program*” (R5). Taken together, these findings suggest that telehealth via mobile-based outreach may be a viable first point of contact for mental health support, with awareness and outreach representing the most immediate barrier to engagement across all service delivery models.

## 4. Discussion

This qualitative study explored the basic and health-related needs of adults with mobility disabilities living in low-resource and rural communities in Alabama, to identify factors that may inform future interventions that address the needs of low-resource, underserved communities of people living with a disability. Participants described constraints that limited health, independence, and community participation, with the most limiting needs revolving around safety, access to mental health support, and barriers to healthy eating, including transportation and affordability. These findings align with national research showing that people with disabilities experience higher rates of poverty, delayed care, reduced access to preventive services, and poorer health outcomes compared to those without disabilities [1–5]. In rural areas, adults with disabilities report both financial and nonfinancial barriers to care at significantly higher rates than rural adults without disabilities [18]. Within the Deep South, disability prevalence is disproportionately concentrated in rural counties with elevated poverty, proving to have significant barriers to routine care and health maintenance [7,8,14]. The findings of this analysis, alongside the previous studies mentioned, reinforce that health behaviors are not just shaped by individual choice, but by whether basic needs and environmental supports are reliably met.

### 4.1. Food Access, Cost, and Nutrition

Food insecurity emerged as a significant limitation on participants’ ability to support their health. Participants described reliance on food banks and limited access to nearby grocery stores offering affordable, healthy options, including concerns about expired or spoiled food within the food banks. Households that include a person with a disability are known to experience higher rates of food insecurity and are more likely to face tradeoffs between food and healthcare-related expenses [19]. In the Southeast, spatial analyses demonstrate a substantial geographic overlap between disability prevalence and food insecurity, suggesting that these challenges are structurally driven rather than individual in nature [20]. The current findings extend this literature by illustrating how food insecurity narrows dietary choice and heightens risk for chronic disease among adults already managing disability-related health conditions.

### 4.2. Mental Health Needs and Absence of Formal Support

Mental health challenges were common across participants and were frequently described as worsening due to limited access to counseling, support groups, and mental health services. National surveillance data indicate that adults with disabilities experience significantly higher rates of frequent mental distress compared to adults without disabilities [21]. These disparities were further exacerbated during the COVID-19 pandemic, when adults with disabilities reported substantially higher rates of anxiety, depression, and suicidal ideation [22]. In this study, participants linked mental health distress to chronic pain, social isolation, and constrained mobility, while also describing the absence of formal mental health supports. Provider shortages and long travel distances with lack of transportation services in rural areas further limit access to behavioral health services, making alternative delivery models increasingly relevant [7,8,18].

### 4.3. Safety and Environmental Barriers to Participation

Participants described environmental safety concerns as barriers to neighborhood engagement, physical activity, and social participation. Fear related to loose dogs, neighborhood safety, and poorly maintained infrastructure discouraged walking and use of community spaces, particularly for individuals with mobility impairments. Prior qualitative research conducted in low-resource communities in Alabama similarly identified unsafe and inaccessible environments as major constraints on community participation for people with disabilities [23]. These findings suggest that participation restrictions are not solely attributable to impairment, but instead emerge from the interaction between disability and unsafe or neglected environments.

### 4.4. Physical Health Deprioritized by Basic Survival Needs

Although participants frequently reported chronic health conditions, physical health maintenance was often deprioritized in favor of meeting immediate survival needs such as food, housing, and transportation. This aligns with evidence showing substantially higher healthcare expenditures among people with disabilities, with costs increasing dramatically as assistance with activities of daily living increases [6]. When individuals must choose between necessities and assistive devices or preventive care, physical health behaviors become secondary, resulting in delay of care [1,2,6].

### 4.5. Implications for Practice and the Field

These findings suggest that improving health equity for people with disabilities in low-resource Deep South communities requires interventions that address basic needs as prerequisites for health. For people with access to the internet, telehealth may be an ideal method for delivering digital services that can address the needs identified in this paper. Telehealth has been shown to reduce travel burden and missed appointments, particularly in rural settings where transportation barriers are common [18,24]. Telehealth also expands access to mental and behavioral health services when in-person care is limited [25]. Additionally, virtual care may improve flexibility and continuity for people with disabilities by reducing travel stress and time-related burdens, provided accessibility needs are addressed [2,26]. Other strategies such as transportation assistance, food access initiatives, and partnerships with trusted community organizations may further support engagement [20,23]. Despite the promise of telecommunications for addressing health, internet access and use is generally lower among people with disabilities and people living in rural or low-resource areas [27–29], and these disparities must be considered when designing interventions that help the most-difficult-to-reach. For example, the Lifeline program (government-funded) provides a monthly discount on broadband or phone services for certain populations. Moreover, providing services with high-perceived value and incorporating user-centered designs are potential solutions to increase telehealth adoption among people with disabilities [30], but far more research is needed to identify solutions that can make a real-world change in the lives of people in need.

Health professionals serving people with mobility disabilities in low-resource communities can take practical steps informed by the findings of this study. Participants consistently described physical health as deprioritized behind survival needs such as food and housing, suggesting that health professionals who screen for or help address unmet basic needs during clinical encounters may see stronger engagement. Awareness of transportation barriers, low-cost internet access, and maintenance of updated referral lists for local community resources are low-burden strategies that could improve care continuity for people with disabilities in underserved areas. Health professionals should also take an active role in connecting people with mobility disabilities to mental health services, as participants described being turned away from referrals despite persistent requests for support. Given that mental health distress was the most urgent need reported in this study and that structural barriers limit this population’s ability to seek help independently, clinical encounters represent an underutilized opportunity to reduce the gap between need and access. Policy efforts should also prioritize expanding telehealth reimbursement for mental health and supportive care services in low-resource communities, as cost was a consistent barrier to accessing care among participants. Without reimbursement structures that account for the financial constraints faced by people with disabilities living in poverty, the promise of telehealth as an access strategy will remain unrealized for those who need it most.

Translating these findings into practice requires concrete starting points. Participants described poor awareness of available resources as a primary barrier to engagement, suggesting that outreach is as important as the services themselves. Community health workers and trusted local organizations are well positioned to close this awareness gap through in-person outreach, given that participants responded to relational rather than digital communication. Device loan programs, public access kiosks, and federal affordability programs represent practical infrastructure solutions for communities with limited internet access. Telehealth Resource Centers, which are federally funded and regionally distributed, offer technical assistance that could help community organizations and health systems extend services into underserved areas. Where state regulations permit, audio-only telehealth options can further reduce technology barriers. Local partnerships between health systems, community organizations, and disability-serving agencies are needed to coordinate these efforts and ensure that the needs identified by participants, including mental health support, food access, and transportation, are addressed in a way that reflects the lived realities of people with disabilities in low-resource communities.

Several limitations should be considered when interpreting these findings. The sample was small and recruited through convenience sampling, which limits generalizability beyond low-resource communities in the Birmingham metropolitan area. Participants were predominantly African American and female, and findings may not reflect the experiences of men or people from other racial and ethnic backgrounds living with mobility disabilities in the Deep South. The study relied on a single phone interview per participant, which limited the depth of information that could be gathered compared to in-person or longitudinal methods. The interview guide was broad by design, which constrained the level of detail captured on any single topic, including telehealth readiness and specific service preferences. Additionally, participants were not screened for prior telehealth experience, and responses regarding remote service delivery may therefore reflect hypothetical attitudes rather than informed experience. Finally, disability status was not verified through clinical records, which introduces the possibility of misclassification.

## 5. Conclusions

Mental health, food insecurity, housing, and physical health care are critical “needs” that impede people from living well with a disability. These needs are unable to be addressed due to financial and community constraints. Considering that low-resource communities are often unsafe, remote services and use of mobile phones for getting on the internet could be a viable solution for addressing needs, and this warrants investigation. The foundational needs identified by participants, including mental health support, food access, and safe community environments, warrant consideration in the planning and delivery of health and telehealth services for people with disabilities in low-resource communities in the Southeast, and point to areas where further investigation is needed.

## Supplementary Material

Supplemental Material 1

**Supplementary Materials:** The following supporting information can be downloaded at: https://www.mdpi.com/article/10.3390/disabilities6020040/s1, Supplementary File S1: Qualitative analysis of interviews using Braun and Clarke’s six-phase thematic analysis approach.

## Figures and Tables

**Table 1.** Participant characteristics

ID	Age	Sex	Ethnicity	Mobility Disability
1	57	F	Mexican and French	Spinal disorder
2	52	F	Native American Indian	Arthritis and comorbidity
3	61	F	African American	Osteoarthritis
4	60	F	Caucasian	Diabetic, fibromyalgia, and neuropathy
5	58	F	African American and Puerto Rican	Obesity and arthritis
6	60	M	African American	Arthritis
7	70	F	African American	Kidney failure
8	58	F	African American	Lower extremity (non-specified)
9	59	F	African American	Lower extremity (non-specified)
10	Unknown	M	African American	Lower extremity (non-specified)
11	56	F	African American	Spinal Stenosis
12	75	F	African American	Lower extremity (non-specified)
13	38	F	African American	Lower extremity (non-specified)
14	78	F	African American	Lower extremity (non-specified)
15	59	F	African American	Spina bifida

Participants responded ‘yes’ when asked if they had a disability that impaired their ability to walk, climb steps, or required use of an assistive device to ambulate.

**Table 2:** . Qualitative analysis of interviews using Braun and Clarke’s six-phase thematic analysis approach.

Theme	Codes	Supporting Quotes
Physical disability and mental illness prevents employment and participation in desirable activities	-Most unemployed and living on disability payments -Physical disability makes it difficult to be employed -Mental illness and physical limitations are linked with home isolation	“My thoughts about my health is that I would say I’m in good health for 70 years old… With afew exceptions of, and the only thing that really has bothered me is my shoulder. I’ve been dealing with it for about a year and a half now and it can’t seem to find out what’s causing all this pain. And that’s despite being a diabetic for ever since I was 20 years old and renal failure and a kidney transplant. I don’t have any problem with any of that stuff. It’s just my shoulder keeps me from doing what I really want to do because I can’t hardly use my right hand.” – R7 “I spend a lot of time in bed, unfortunately. Part of it’s depression, but 80% of it is pain.”R1
Neglected by the larger, external community	-Perceive themselves to be exterior to greater city areas -Neighborhood infrastructure does not receive regular maintenance -Insufficient first responders for the needs of the area -Cost of living increasing compared to what they can afford	“I think people forget about us up here on this hill. You know, we kind of off the forbidden path, so to speak. So I think they forget about us up here because a lot of times I have to call the city for to cut weeds across the street and all that. They just forget about us.” R7 “ Well, I think, I think that I’m a representative in this area and I think I’ve seen on next door and stuff like that, that people have contacted him with no avail. They also contacted the male’s office with no avail. So I really don’t know what we could do. I really don’t.” R7 “You know, so just like I say, it might just be that we, we have forgotten because sometimes the garbage don’t, just like we have garbage served their money. I had to call about a month ago. They didn’t even pick the garbage up. It was sitting out there for two, three days, but I’m thinking it happened because now they sent them right on when I called 3-1-1, but I’m thinking it happened because they just forget about us.” R7 “No, nothing that I could think of right now, but I’ll tell you what, another thing yesterday or something, I think they should have, like I said, you know, I told you we all been there. They need to have the police to ride up to him more, you know, they need, I don’t ever see them come take that fire hydrant. They need to make sure these fire hydrants work. One of my neighbor’s house caught a fire probably about six, seven years ago. And I just, when I came home from work, I was working in, they had a hose ran all the way up the street. I mean, it was all the way up the hill and a fire hydrant right across the street from my house. And I lived two houses from the neighbor, but they didn’t know it was up here. See, stuff like that to me don’t make no sense. Cause the fire department should know where every fire hose is. So it’s just stuff like that that just don’t make sense to me. If I was a neighbor, I would have sued the city. I don’t know what she did cause I hadn’t asked her. But that, you know, Toronto and we couldn’t even get up here. We couldn’t drive up here. We had to leave our car and pull over the hill and walk to see what was going on. To me, stuff like that don’t make sense. So it’s just, they need to not file any conclusion. They just need to make sure that this little area up here, this circle or whatever they want to call it, it’s not forgotten about because I feel it here.” R7 “We don’t have sidewalks. We do not. And I think we need sidewalks. Not only do we need sidewalks, we need a couple of speed bumps”R6
Abundance of spirituality resources that help maintain mental health	-Churches readily available -Beliefs in faith provide resilience -Faith is part of the culture -Being around others at church creates community and social opportunity	“It’s churches on every corner. Let me just say that. I don’t go to church on this side of town. I go to church on way where I went, been going here since I was 14, way on the other side of town. So, but I’m sure a lot of, it’s a lot of churches over here and you know, I’m quite sure if I wanted to, it would meet my spiritual needs.”R7 “So, you know, I understand that I look to the hill, which caused my health, you know, God, you know, He’ll sit with you in a, you know, in a gathering, you know what I’m saying? Like you said, it’d be two or three, you know, more in the midst, God in the midst. So I understand that He’s going to sit with you if you’re by yourself. But at the same time, you know, I don’t understand when you’re going into like a spiritual thing, you want to be around people.R7 “Yeah, I mean, like, starting out, like, in my community, it needs to be more focused on the road. Because like I said, people can fall down because there’s a lot of potholes. And if you get people like me with, you know, like, mobility, and it’s like in my handicap, you know, saying for my disability, it can really, really hurt me if I come to those, you know what I’m saying, situations where I’m like that.”R6 “As far as me personally, you know, my mental health is pretty decent. You know, a lot of it has to do with my family and friends. I have a really good support team.”R5 “I’ve been through some struggles, been through some heartache and pain or whatever, death or whatever you want to call it, like everybody else. But as long as you put God first, everything will be all right.”R3
Uncontrollable dangers prevent participation outdoors	-Poverty creates desperation and crime, which limits local networking -Loose dogs perceived as a serious threat towards health and safety -Community spaces are not safe due to violence and loitering -Inaccessible built-environment (e.g., sidewalks) creates participation hazards -Mental disorders in community members not well managed	“So I’d be real mindful of my situation and where I’m going. Like I said, you really can’t enjoy like, around the block walk, because I know dogs come and they want to take over the neighborhood and everything.”-R6 “Well, they got a lot of stray dogs and stuff around here. You know, they need to put their dogs up because it makes you scared to walk because you know, you hear every day about dogs attacking people and killing them. Otherwise the neighborhood is quiet. You know, we don’t have a lot of excitement over here. Well, we once did because we had a couple of murders over here on my street, but you know, it’s, uh, otherwise, you know, it’s fine. We don’t have any, you know, big time problems over here.” R7 “The closest park to me is East Lake. It’s right down. I don’t live far from East Lake Park. And, uh, you know, they claimed in a runaway that I hadn’t been down there cause they always got something going on if somebody getting shot or something. And I just don’t feel safe. I’ve heard that. That’s another thing too. I feel like it’s a lot of safety issues these days too. People want to get out, but then are they safe?” R7 “Sometimes community things you know you let people in just a little bit and they take you give them a little inch and take them out so it is kind of tough to trust people around your house or in community settings. “R6 “A lot of times people like when things ain’t going their way, they should just go steal, rob, hurt somebody or whatever.”R6 “We don’t have sidewalks. We do not. And I think we need sidewalks. Not only do we need sidewalks, we need a couple of speed bumps cause by this being a one way in and one way out, some of these youngsters come up through here, they drive so fast. We don’t have many young children in the neighborhood, but we do have older people that walk, you know”R6 “No, I don’t think my neighborhood is accessible for that. All the porches are high. The steps are deep.”R5
Mental health is a severe issue that is worsened by a lack of support (counselling and socialization)	-Depression was common and debilitating -Biopolar and other disorders were common in participants and others in their community - Counselling support to manage mental health is highly desired -Counselling support inaccessible (high cost) -Stress regarding safety in the community negatively affected mental health -Lack of socializing with others negatively impacted mental health	“Yes, my mental health, you know, it’s a lot of things. It’s hard when you’re dealing with people because everybody has these personalities.” Raven 6 “mental health, you know, they have not, what is it, healed that inner child or whatever it is, whatever is going on, it stems from the way we grew up. And if you have not healed from whatever it is, whatever trauma or, what’s the word, not trauma, but…um dysfunction, and it’ll manifest in your adulthood, good, bad or ugly, so.”R3 “The one thing they don’t have, I wish they did, is I also deal with depression and anxiety and I have bipolar. So, they don’t offer, no recreation support groups to basically support my mental health. There’s not no support group out there for people that are like me, that have to deal with these problems every day.”R2 “Well, one thing they could assist me with and I found, like I said, the main thing is my mental health is because there is young kids out here that they don’t have no recreation programs. I’m talking about art classes, you know, in the liberal arts because people are taking that liberal arts is not important, but it is. Because it’s reached into the medical health field now that it’s used as therapy, art therapy for mental illness. And I wish they would have, you know, somebody that is qualified that can teach art classes and give art therapy classes here. I think it would really help these teens of not wanting to go into the streets and get into trouble or get hurt. The only thing that helped me really feel included is the support that I’m not getting as far as them just opening up their minds to, like I said, getting the kids more involved and off the streets. “R2 “ I wish that there was more resources for mental illness and, you know, the kids to get the help they can. People just reaching out, reaching out. Put yourself in our shoes and see what exactly we go through.”R2 “I spend a lot of time in bed, unfortunately. Part of it’s depression, but 80% of it is pain.”R1 “I just don’t have any social life. So I cut everybody out of my life. I was way too emotional for the first year. I couldn’t even speak without crying.”R1 “If there was to provide the funds, I think they should target it on opening up counseling support groups. A lot of people here really need the counseling support. That is the only thing that I see is really liking in the library that should be open. Because there’s a lot of young mothers here that they don’t have the financial support as well as the emotional support. I’m just feeling like they’re being a good mother.”R2
High cost and inefficient public transportation limits outside activities to basic needs	-High cost of gasoline -No available car or no driver’s license -Public transportation not suitable for some needs -Public transportation not convenient -Rely on family members for transportation -Burdensome reliance on others for transportation -Transportation requires complex planning -Travel outside of the home only done for necessary activities (grocery store, doctor’s appointments, and church)	“I ain’t have transportation. I ain’t have no gas. You know I ain’t have anywhere something like that but you know it’s gonna give you like a like a gas card, a gift card”R6 “Well, what we need is some kind of transportation for the elderly people that don’t drive and like me, don’t drive. And when you don’t have the gas to get around to have somebody to come and help us like go to the store or get to the doctor. I’ve had to cancel out many doctor appointments because I’ve not had a way of getting to them.”R4 “Um, the buses could run a little bit longer and they don’t run long enough and they, they cut off like, say like we want to go to a store or whatever. Let’s just say I want to go to church on Sunday and my church is in Hoover. Okay. And I stay in Fairfield. I can’t get there. So more accessibility with the public transportation and, um, they have an orange bus, but the orange bus only goes certain places. And to me it’s just a waste of money.”R3 “I could try to go to again, but I feel like that’s asking my family to just have to do another thing for me.”R1
High cost of food creates a reliance on foodbanks and limits access to healthy eating choices	-Food bank use was common -Food bank use necessary to sustain themselves and family members -Food is too expensive at major grocery chain stores -Healthy food perceived to be too expensive -Transportation difficulties (cost and access) limited choices to nearby grocery stores -Not aware of how to gain access to a local food bank	“I have this thing about a senior citizen garden. I don’t know why, but I’ve been thinking about it and wondering about it with the price of food and everything nowadays. It would be nice to have a community garden and that would be something for senior citizens to work on, to get out of the house to exercise, you know.”R5 “I haven’t seen any and I have a couple of lists for the food banks because I do check them out. I haven’t seen any in this area. I don’t see any type of food vans or anything like that, meals for wheels or nothing like that for senior citizens coming through or coming around.”R5 “Well, our uh, our food bank that that I went to the first time when I got over here, where I’m at now, they give us food. That was, it was all outdated. It’s like two years outdated. I do not eat a food a year outdated. They gave me a key lime pie, and it was nearly a year outdated. And give me a heap of cabbage. It was when I cut into it, it was black on the inside. “R4 “I think we need a grocery store in our neighborhood. Now I stay in the rough part, but I don’t care. I’ve always stayed in the rough part, but you know, it’s what you make it. It’s not the neighborhood, it’s the people in the neighborhood. You know, so basically we need a grocery store that’s reasonable, that sells food that we, healthy food.”R3 “Yes, as far as we also have a food bank that is very much met. That has helped a lot of people that are in need of food. Around here and it has really been a successful. I do very much thank God for that.”R2 “We have food pantries and stuff. And see, I’ve always been a go-getter, you know, even before the food pantries became popular. I’ve always, you know, by me having a child and she having her friends, you know, it’s like teenagers are bottomless pits. So I’d be like, listen, I’m going to this food bank over here. I’ll make a big old pot of spaghetti. Y’all do what you want. And you got noodles, you got sandwich makings. And my mom used to say on Friday, listen, you on your own. Everybody for themselves. You know, you big enough, I’ll make you a salad or a sandwich or whatever, oatmeal, whatever.”R3
Limited participation in programs to improve physical health	-Costs related to healthcare are unmanageable due to high cost of living -Cardiometabolic disease is prevalent and a daily concern -Access to therapy or health-related visits was limited due to transportation -Physical health was perceived as a secondary concern to basic needs (food and mental health)	“Well, no, not where I’m at.”R4 “We need insurance for our body. And I feel like insurance for our body is the resources, making it readily accessible and putting it out there, just like we do everything else, you know, make it a priority, basically, and make it something almost like mandatory, you know, because where I live, we have to have mandatory insurance on our apartment. So why can’t we have mandatory exercise?”R3 “Despite being a diabetic for ever since I was 20 years old and renal failure and a kidney transplant.”R7 “Well, no, I don’t feel like I do have that much because there is, our library down here does exercise and, you know, with aerobics and they do other programs that are offered, but it’s still not enough. They still need to improve on that. Like they don’t have, um like, aerobics instructors or aerobics classes dealing with aerobics. This is just a class that is called chair exercise that they’re using a chair to do basic exercises for older people. They don’t have nobody to really target the young people like me that are disabled.” R2 “No, I live in X and there’s just not very much going, you know, like there’s no parks. I think it’s underfunded. More parks, more places to walk. Even when I was roller skating, I would roller skate outdoors and I had to drive miles to find like a greenway or there’s just not much out here. It’s not taken care of very well.”R1 “I’ve been trying to get help, help getting a $300 to get me some hearing aids. And I was having to pay what money my husband brings in to first have a roof over our heads, for my two children. And I don’t have $300 for no hearing aids.”R4 “Because they can’t get my sugar under control, my blood pressure stays high.”-R4 “Like in the days, I really can’t because my diabetes, if it’s a certain temperature, I will pass out. I can’t take the heat.”-R2

## Data Availability

The data beyond what is provided in this manuscript are not publicly available due to privacy concerns.
