# Supplementary material for: Reaching the Unreached: Unmet Needs and the Promise of Telehealth Among People with Mobility Disabilities in Low-Resource Areas in Alabama
Source: Disabilities (Basel). Author manuscript; Available in PMC 2026 Jul 2. (PMC13322284; doi:10.3390/disabilities6020040)
Supplement: Supplemental Material 1 [file NIHMS2188124-supplement-Supplemental_Material_1.docx]

**Appendix 1. Qualitative analysis of interviews using Braun and Clarke’s six-phase thematic analysis approach.**

| **Theme** | **Sub-Theme** | **Codes** | **Supporting Quotes** |
| --- | --- | --- | --- |
| Seeking Stability Amid Severe Mental Health Strain and Inadequate Supports | Mental health decline in the absence of reliable social support | Mental illness and physical limitations are linked with home isolation  Lack of socializing with others negatively impacted mental health  Social networks were small  Participants often did not have strong connections to family members | “I spend a lot of time in bed, unfortunately. Part of it's depression, but 80% of it is pain.”R1  “Yes, my mental health, you know, it's a lot of things. It's hard when you're dealing with people because everybody has these personalities.”  Raven 6  “mental health, you know, they have not, what is it, healed that inner child or whatever it is, whatever is going on, it stems from the way we grew up. And if you have not healed from whatever it is, whatever trauma or, what's the word, not trauma, but...um dysfunction, and it'll manifest in your adulthood, good, bad or ugly, so.”R3  “The one thing they don't have, I wish they did, is I also deal with depression and anxiety and I have bipolar. So, they don't offer, no recreation support groups to basically support my mental health. There's not no support group out there for people that are like me, that have to deal with these problems every day.”R2  “Well, one thing they could assist me with and I found, like I said, the main thing is my mental health is because there is young kids out here that they don't have no recreation programs. I'm talking about art classes, you know, in the liberal arts because people are taking that liberal arts is not important, but it is. Because it's reached into the medical health field now that it's used as therapy, art therapy for mental illness. And I wish they would have, you know, somebody that is qualified that can teach art classes and give art therapy classes here. I think it would really help these teens of not wanting to go into the streets and get into trouble or get hurt. The only thing that helped me really feel included is the support that I'm not getting as far as them just opening up their minds to, like I said, getting the kids more involved and off the streets.  ”R2  “ I wish that there was more resources for mental illness and, you know, the kids to get the help they can. People just reaching out, reaching out. Put yourself in our shoes and see what exactly we go through.”R2  “I spend a lot of time in bed, unfortunately.  Part of it's depression, but 80% of it is pain.”R1  “I just don't have any social life. So I cut everybody out of my life. I was way too emotional for the first year. I couldn't even speak without crying.”R1  “If there was to provide the funds, I think they should target it on opening up counseling support groups. A lot of people here really need the counseling support. That is the only thing that I see is really liking in the library that should be open. Because there's a lot of young mothers here that they don't have the financial support as well as the emotional support. I'm just feeling like they're being a good mother.”R2 |
|  | Mental health is a severe issue that is worsened by a lack of professional support | Depression was common and debilitating  Biopolar and other disorders were common in participants and others in their community  Counselling support to manage mental health is highly desired  Counselling support inaccessible (high cost)  Stress regarding safety in the community negatively affected mental health |  |
|  | Spirituality: abundant and supportive but insufficient buffer for well-being | Churches readily available  Beliefs in faith provide resilience  Faith is part of the culture  Being around others at church creates community and social opportunity | “It's churches on every corner. Let me just say that. I don't go to church on this side of town. I go to church on way where I went, been going here since I was 14, way on the other side of  town. So, but I'm sure a lot of, it's a lot of churches over here and you know, I'm quite sure if I wanted to, it would meet my spiritual needs.”R7  “So, you know, I understand that I look to the hill, which caused my health, you know, God, you know, He'll sit with you in a, you know, in a gathering, you know what I'm saying? Like you said, it'd be two or three, you know, more in the midst, God in the midst. So I understand that He's going to sit with you if you're by yourself. But at the same time, you know, I don't understand when you're going into like a spiritual thing, you want to be around people.R7  “Yeah, I mean, like, starting out, like, in my community, it needs to be more focused on the road. Because like I said, people can fall down because there's a lot of potholes. And if you get people like me with, you know, like, mobility, and it's like in my handicap, you know, saying for my disability, it can really, really hurt me if I come to those, you know what I'm saying, situations where I'm like that.”R6  “As far as me personally, you know, my mental health is pretty decent. You know, a lot of it has to do with my family and friends. I have a really good support team.”R5  “I've been through some struggles, been through some heartache and pain or whatever, death or whatever you want to call it, like everybody else. But as long as you put God first, everything will be all right.”R3 |
| Constrained food environments shaped by cost, location, and safety | Government support maintains the cost of living through food access | Food bank use was common, but the food quality was poor  Food bank use necessary to sustain themselves and family members | “I have this thing about a senior citizen garden. I don't know why, but I've been thinking about it and wondering about it with the price of food and everything nowadays. It would be nice to have a community garden and that would be something for senior citizens to work on, to get out of the house to exercise, you know.”R5  “I haven't seen any and I have a couple of lists for the food banks because I do check them out. I haven't seen any in this area. I don't see any type of food vans or anything like that, meals for wheels or nothing like that for senior citizens coming through or coming around.”R5  “Well, our uh, our food bank that that I went to the first time when I got over here, where I'm at now, they give us food. That was, it was all outdated. It's like two years outdated. I do not eat a food a year outdated. They gave me a key lime pie, and it was nearly a year outdated. And give me a heap of cabbage. It was when I cut into it, it was black on the inside.  ”R4  “I think we need a grocery store in our neighborhood. Now I stay in the rough part, but I don't care. I've always stayed in the rough part, but you know, it's what you make it. It's not the neighborhood, it's the people in the neighborhood. You know, so basically we need a grocery store that's reasonable, that sells food that we, healthy food.”R3  “Yes, as far as we also have a food bank that is very much met. That has helped a lot of people that are in need of food. Around here and it has really been a successful. I do very much thank God for that.”R2  “We have food pantries and stuff. And see, I've always been a go-getter, you know, even before the food pantries became popular. I've always, you know, by me having a child and she having her friends, you know, it's like teenagers are bottomless pits. So I'd be like, listen, I'm going to this food bank over here. I'll make a big old pot of spaghetti. Y'all do what you want. And you got noodles, you got sandwich makings. And my mom used to say on Friday, listen, you on your own. Everybody for themselves. You know, you big enough, I'll make you a salad or a sandwich or whatever, oatmeal, whatever.”R3 |
|  | The cost of food at grocery stores is increasing beyond their means | High cost of food at grocery stores heavily impacts cost of living  Healthy food restricted by high cost |  |
|  | Food options limited to those nearby | Access to food is limited by public transportation  Cost of gas limited community participation |  |
|  | Walking or wheeling to grocery stores perceived as unsafe | No sidewalks in the community  Road conditions not well kept and unsafe |  |
| Feeling forgotten: Systemic neglect and restricted participation in community life | Neglected by the larger, external community | Perceive themselves to be exterior to greater city areas  Neighborhood infrastructure does not receive regular maintenance  Insufficient first responders for the needs of the area  Cost of living increasing compared to what they can afford | “I think people forget about us up here on this hill. You know, we kind of off the forbidden path, so to speak. So I think they  forget about us up here because a lot of times I have to call the city for to cut weeds across the street and all that. They just forget about us.” R7  “ Well, I think, I think that I'm a representative in this area and I think I've seen on next door and stuff like that, that people have contacted him with no avail. They also contacted the  male's office with no avail. So I really don't know what we could do. I really don't.” R7  “You know, so just like I say, it might just be that we, we have forgotten because sometimes the garbage don't, just like we have garbage served their money. I had to call about a month ago. They didn't even pick the garbage up. It was sitting out there for two, three days, but I'm thinking it happened because now they sent them right on when I called  3-1-1, but I'm thinking it happened because they just forget about us.” R7  “No, nothing that I could think of right now, but I'll tell you what, another thing yesterday or something, I think they should have, like I said, you know, I told you we all been there. They need to have the police to ride up to him more, you know, they need, I don't ever see them come take that fire hydrant. They need to make sure these fire hydrants work.  One of my neighbor's house caught a fire probably about six, seven years ago. And I just, when I came home from work, I was working in, they had a hose ran all the way up the  street. I mean, it was all the way up the hill and a fire hydrant right across the street from my house. And I lived two houses from the neighbor, but they didn't know it was up here.  See, stuff like that to me don't make no sense. Cause the fire department should know where every fire hose is. So it's just stuff like that that just don't make sense to me. If I was a  neighbor, I would have sued the city. I don't know what she did cause I hadn't asked her. But that, you know, Toronto and we couldn't even get up here. We couldn't drive up here. We  had to leave our car and pull over the hill and walk to see what was going on. To me, stuff like that don't make sense. So it's just, they need to not file any conclusion. They just need  to make sure that this little area up here, this circle or whatever they want to call it, it's not  forgotten about because I feel it here.” R7  “We don't have sidewalks. We do not. And I think we need sidewalks. Not only do we need  sidewalks, we need a couple of speed bumps”R6 |
|  | Uncontrollable dangers prevent participation outdoors | Poverty creates desperation and crime, which limits local networking  Loose dogs perceived as a serious threat towards health and safety  Community spaces are not safe due to violence and loitering  Inaccessible built-environment (e.g., sidewalks) creates participation hazards  Mental disorders in community members not well managed | “So I'd be real mindful of my situation and where I'm going. Like I said, you really can't enjoy like, around the block walk, because I know dogs come and they want to take over the neighborhood and everything.”-R6  “Well, they got a lot of stray dogs and stuff around here. You know, they need to put their  dogs up because it makes you scared to walk because you know, you hear every day about  dogs attacking people and killing them. Otherwise the neighborhood is quiet. You know, we don't have a lot of excitement over here. Well, we once did because we had a couple of murders over here on my street, but you know, it's, uh, otherwise, you know, it's fine. We don't have any, you know, big time problems over here.” R7  “The closest park to me is East Lake. It's right down. I don't live far from East Lake Park. And, uh, you know, they claimed in a runaway that I hadn't been down there cause they always got something going on if somebody getting shot or something. And I just don't feel safe. I've heard that. That's another thing too. I feel like it's a lot of safety issues these days too. People want to get out, but then are they safe?” R7  “Sometimes community things you know you let people in just a little bit and they take you give them a little inch and take them out so it is kind of tough to trust people around your house or in community settings. ”R6  “A lot of times people like when things ain’t going their way, they should just go steal, rob, hurt somebody or whatever.”R6  “We don't have sidewalks. We do not. And I think we need sidewalks. Not only do we need sidewalks, we need a couple of speed bumps cause by this being a one way in and one way  out, some of these youngsters come up through here, they drive so fast. We don't have many young children in the neighborhood, but we do have older people that walk, you  know”R6  “No, I don't think my neighborhood is accessible for that. All the porches are high. The steps are deep.”R5 |
|  | High cost and inefficient public transportation limits outside activities to basic needs | High cost of gasoline  No available car or no driver’s license  Public transportation not suitable for some needs  Public transportation not convenient  Rely on family members for transportation  Burdensome reliance on others for transportation  Transportation requires complex planning  Travel outside of the home only done for necessary activities (grocery store, doctor’s appointments, and church) | “I ain't have transportation. I ain't have no gas. You know I ain't have anywhere something like that but you know it's gonna give you like a like a gas card, a gift card”R6  “Well, what we need is some kind of transportation for the elderly people that don't drive and like me, don't drive. And when you don't have the gas to get around to have somebody to come and help us like go to the store or get to the doctor. I've had to cancel out many doctor appointments because I've not had a way of getting to them.”R4  “Um, the buses could run a little bit longer and they don't run long enough and they, they cut off like, say like we want to go to a store or whatever. Let's just say I want to go to church on Sunday and my church is in Hoover. Okay. And I stay in Fairfield. I can't get there. So more accessibility with the public transportation and, um, they have an orange bus, but the orange bus only goes certain places. And to me it's just a waste of money.”R3  “I could try to go to again, but I feel like that's asking my family to just have to do another thing for me.”R1 |
| Physical Health Deprioritized by Structural Barriers and Competing Needs | Structural barriers limit access to health care and health-promoting activities | Costs related to healthcare are unmanageable due to high cost of living  Access to therapy or health-related visits was limited due to transportation  Limited participation in programs to improve physical health  Limited local resources for exercise participation | “Well, no, not where I'm at.”R4  “We need insurance for our body. And I feel like insurance for our body is the resources, making it readily accessible and putting it out there, just like we do everything else, you know, make it a priority, basically, and make it something almost like mandatory, you know, because where I live, we have to have mandatory insurance on our apartment. So why can't we have mandatory exercise?”R3  “Despite being a diabetic for ever since I was 20 years old and renal failure and a kidney transplant.”R7  “Well, no, I don't feel like I do have that much because there is, our library down here does exercise and, you know, with aerobics and they do other programs that are offered, but it's still not enough. They still need to improve on that. Like they don't have, um like, aerobics instructors or aerobics classes dealing with aerobics. This is just a class that is called chair exercise that they're using a chair to do basic exercises for older people. They don't have nobody to really target the young people like me that are disabled.” R2  “No, I live in X and there's just not very much going, you know, like there's no parks. I  think it's underfunded. More parks, more places to walk. Even when I was roller skating, I would roller skate outdoors and I had to drive miles to find like a greenway or there's just not much out here. It's not taken care of very well.”R1  “I've been trying to get help, help getting a $300 to get me some hearing aids. And I was having to pay what money my husband brings in to first have a roof over our heads, for my two children. And I don't have $300 for no hearing aids.”R4  “Because they can't get my sugar under control, my blood pressure stays high.”-R4  “Like in the days, I really can't because my diabetes, if it's a certain temperature, I will pass out. I can't take the heat.”-R2 |
|  | Physical health was a substantial burden but was perceived as a secondary concern to competing needs (food and mental health) | Cardiometabolic disease is prevalent  Chronic health conditions are a daily concern that impede community participation  Physical health not identified as a need, despite physical challenges affecting activities of daily living |  |
